# Stroke patients and caregivers’ experiences and satisfaction with post-discharge self-management support in Ghana: A qualitative study

**DOI:** 10.1371/journal.pgph.0006702

**Published:** 2026-06-26

**Authors:** Rockson Ansong, Priscilla Gazarian, Michelle Danny Stampley Boakye, Evans Kyei, Grace Kyei, Eric Oduro, Lingling Zhang

**Affiliations:** 1 Manning College of Nursing and Health Sciences, University of Massachusetts Boston, Boston, Massachusetts, United States of America; 2 Department of African and African American Studies, Louisiana State University, Baton Rouge, Louisiana, United States of America; 3 Tan Chingfen Graduate School of Nursing, University of Massachusetts Chan Medical School, Worcester, Massachusetts, United States of America; 4 Capstone College of Nursing, The University of Alabama, Tuscaloosa, Alabama, United States of America; 5 University Hospital, Kwame Nkrumah University of Science and Technology, Kumasi, Ashanti Region, Ghana; PLOS: Public Library of Science, UNITED STATES OF AMERICA

## Abstract

Self-management support (SMS) is essential for improving stroke recovery and quality of life, yet Ghana faces significant challenges in post-discharge care delivery. Despite high stroke mortality rates after hospital discharge in sub-Saharan Africa, limited research has examined the experiences of stroke survivors and caregivers with healthcare provider support during the transition to home-based management. Understanding these experiences is crucial for identifying gaps and developing targeted interventions. Therefore, this study aimed to explore stroke survivors’ and caregivers’ experiences with post-discharge self-management support from healthcare providers in Ghana, including the adequacy, perceived impact, and satisfaction with existing support systems. Using Thorne’s Interpretive Description approach, data were collected through fifteen dyadic interviews with stroke survivors and their primary caregivers. Data collection and analysis occurred concurrently, guided by Thorne’s three-phase thematic analysis framework and informed by the Chronic Care Model to contextualize findings within evidence-based chronic care principles. Two main themes emerged: (1) unmet educational and support needs for home-based self-management, and (2) perceived impact and satisfaction with support system. Participants reported insufficient pre-discharge education, inadequate caregiver training, lack of structured follow-up, overlooked psychosocial and sexual health needs, and limited collaborative goal-setting. These gaps undermined survivors’ confidence in self-management and satisfaction with care, leading some to seek alternative or unqualified guidance. The findings reveal a disconnect between current post-discharge practices in Ghana and established chronic care principles. Addressing these gaps requires targeted structural and policy reforms, enhanced provider training in SMS strategies, and the development of culturally appropriate interventions to meet the complex needs of stroke survivors and their caregivers.

## Introduction

Stroke is a major public health issue worldwide, disproportionately affecting people of African descent. Studies show higher prevalence, mortality, and recurrence rates among Black populations [1], particularly in resource-limited settings [2]. Ghana has been noted as one of the sub-Saharan African countries with the highest number of stroke-related deaths occurring after hospital discharge, exceeding even the mortality rates among inpatients admitted in critical stages of the disease [3,4]. These post-discharge outcomes point to a critical gap in the continuity of care for stroke survivors, particularly in the transition from hospital to home. One promising approach to addressing this gap is the promotion of effective self-management support (SMS) at home.

Self-management is an essential component of chronic disease management, including stroke. It involves an active, ongoing process in which individuals take charge of managing their condition. Effective self-management has been shown to enhance recovery, improve health outcomes, and increase the quality of life for stroke survivors while reducing healthcare costs [5]. However, many stroke survivors face challenges in managing their condition independently due to limited knowledge and skills [6]. Structured SMS from healthcare providers is therefore essential and required for effective home-based disease management. SMS is a patient-centered approach that empowers individuals by enhancing their confidence, competence, and motivation to manage their health conditions more effectively. It extends beyond providing education to include helping individuals identify challenges, set goals, create action plans, and develop problem-solving skills. SMS also involves active collaboration between patients and healthcare providers, coordinated care, continuous evaluation, and attention to patients’ evolving needs [7].

Despite the importance of SMS, Ghana’s healthcare system faces major barriers to providing comprehensive post-discharge care. The absence of formal post-discharge support policies and standardized stroke care frameworks [8,9] leaves providers to rely heavily on their discretion and expertise. This gap leads to inconsistent quality of care and fragmented service delivery that often fails to meet the complex needs of survivors and caregivers. Studies report systemic deficiencies in post-discharge stroke care, including limited multidisciplinary collaboration, suboptimal provider-patient communication, and insufficient follow-up support [10,11]. These deficiencies leave many stroke survivors inadequately prepared for the challenges of home-based recovery, contributing to increased risks of secondary complications, preventable hospital readmissions, and premature mortality [12].

While previous research in Ghana has covered stroke epidemiology, inpatient care, and general post-discharge issues, little is known about self-management support from the perspective of survivors and caregivers. Existing studies have not fully examined the adequacy, quality, and impact of the support providers offer after discharge or whether current practices reflect recommended SMS roles and processes. There is also limited attention to how sociocultural norms and resource constraints shape what survivors and caregivers expect and how they experience support. These gaps leave the specific SMS needs of Ghanaian stroke survivors and how well these needs are being met unclear.

Emerging studies suggest that culturally sensitive, context-specific strategies could improve long-term outcomes and reduce mortality among stroke survivors in Ghana [13]. Developing such approaches requires a deeper understanding of survivors’ and caregivers’ lived experiences with post-discharge support, as well as the specific ways in which SMS fails or succeeds within routine care. This study addresses the existing gaps in the literature by offering a detailed examination of how stroke survivors and caregivers in Ghana perceive the adequacy and impact of SMS provided by healthcare professionals after discharge. It also offers a new contribution by using a recent conceptual framework to systematically map survivors’ and caregivers’ experiences onto the defined roles of healthcare providers in delivering SMS. This provides an opportunity to identify specific gaps between expected and actual support, which have not been documented in prior studies.

This study examines the experiences of stroke survivors and caregivers regarding post-discharge support from healthcare providers, focusing on the adequacy, perceived impact, and satisfaction with this support. By comparing real-world experiences with the framework’s recommended SMS roles, the study identifies unmet needs and priority areas for improvement. The findings aim to inform culturally appropriate, patient-centered strategies to strengthen SMS and improve post-discharge outcomes for stroke survivors in Ghana.

## Materials and methods

### Ethics statement

Ethical clearance for the study was obtained from both the University of Massachusetts Boston Institutional Review Board and the Committee for Human Research and Publication Ethics at the Kwame Nkrumah University of Science and Technology (KNUST) (Approval numbers: 3435 and CHRPE/AP/1251/24, respectively). All participants were thoroughly informed about the research purpose, procedures, and their rights, and provided written informed consent prior to data collection. Strict protocols were followed to ensure confidentiality and data protection. Only authorized members of the research team accessed interview recordings and transcripts, aligning with the highest standards of ethical research practice.

### Design

This study was part of a broader study that explored SMS practices in Ghana. To gain insight into the lived experiences of stroke survivors and their caregivers, the study adopted Thorne’s interpretive description methodology [14]. This research approach is well grounded in the applied health sciences and enables both rich descriptions and thoughtful interpretations of complex health-related experiences from the perspectives of those directly affected. It is particularly well-suited for generating practice-relevant knowledge from experiential data. Applying this methodological approach enabled an in-depth exploration of participants’ stroke recovery journeys and post-discharge care experiences, uncovering nuanced challenges that can guide the development of contextually and culturally appropriate self-management interventions [14].

### Theoretical framework

**Fig 1:**
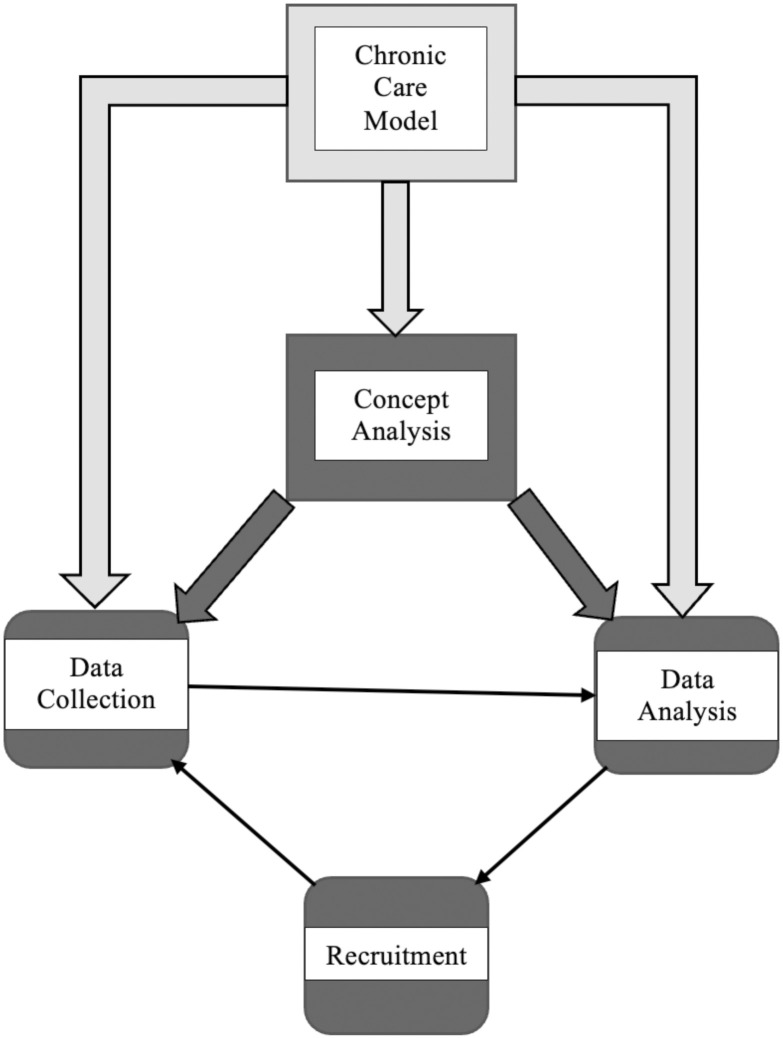
A diagram of the theoretical framework, demonstrating how the Chronic Care Model and the conceptual analysis informed the study.

To effectively identify gaps in current support and understand the needs of stroke survivors and their caregivers, while aligning with chronic disease management principles and ensuring adaptability to Ghana’s healthcare context, this study was guided by both the Chronic Care Model (CCM) and a recent conceptual analysis highlighting key support needs of stroke survivors after hospital discharge [7]. The interview guide (S1 Appendix) was developed using this combined framework. In the absence of a standardized support framework in Ghana, the conceptual framework, informed by the CCM, provided a useful lens for evaluating existing post-discharge support for stroke survivors and their caregivers. This approach helped identify gaps in the support currently provided by healthcare professionals for home-based disease management. Fig 1 illustrates the integration of the CCM and the post-discharge stroke SMS concept analysis, demonstrating how both frameworks informed the data collection and analysis phases of the study.

The CCM offers a comprehensive framework for managing chronic non-communicable diseases, encompassing six interrelated domains: self-management support, healthcare organization, clinical information systems, delivery system design, community resources, and decision support. Central to the CCM is the emphasis on meaningful interactions between informed, engaged patients and healthcare teams that are prepared, proactive, and responsive to patients’ needs [15]. To achieve optimal patient outcomes, these interactions must address the needs of both patients and their families, encouraging active involvement and participation in care [15]. Effective implementation of SMS requires active involvement of both patients and their families, alongside healthcare team members who are equipped to apply SMS principles consistently. In this study, the CCM and the conceptual framework informed not only the development of the interview guide but also the analysis and discussion of findings. This allowed for a structured yet flexible exploration of stroke survivors’ and caregivers’ experiences, enabling a richer understanding of the care processes and system-level factors influencing post-discharge self-management support practices in Ghana.

### Study setting and recruitment

The study was conducted at the Kwame Nkrumah University of Science and Technology (KNUST) Hospital in Ghana. This university-affiliated facility, supported by government funding, provides medical care to both university affiliates and the general public. It operates within Ghana’s broader public health infrastructure, participating in the National Health Insurance Scheme (NHIS), which enhances accessibility for socioeconomically diverse populations. The hospital serves as a secondary care center with capacities for both routine and specialized services, including chronic disease and stroke management [16].

Participants were recruited through purposive sampling, specifically targeting stroke survivors who had been discharged from the hospital and their caregivers. A total of 16 stroke survivors and their caregivers were screened, with one pair excluded because they had been discharged from another facility and were only seeking follow-up care at KNUST Hospital, which did not meet the inclusion criteria. All remaining eligible participants agreed to take part, resulting in a final sample of 30 individuals (15 stroke survivors and their caregivers). The sample size was guided by prior interpretive descriptive studies that examined related topics using a similar number of participants [17], providing sufficient depth of insight while acknowledging that the findings do not provide a comprehensive representation of all possible experiences [18,19].

### Inclusion and exclusion criteria

Stroke survivors were eligible to participate if they were at least 18 years old, had experienced either an ischemic or hemorrhagic stroke, and had been discharged from KNUST Hospital. Additionally, they must have been managing their condition at home for at least two weeks post-discharge. This timeframe ensured they had sufficient self-management experience to provide informed insights into their post-discharge care and challenges [20]. Participants also needed the legal capacity to provide informed consent. Given the critical role of caregivers in stroke self-management, this study only included participants who had a primary caregiver. A primary caregiver could be a family member, friend, or paid caregiver, primarily responsible for supporting their self-management. Including caregivers was essential to gaining a holistic perspective on stroke care, highlighting both their experiences and the support strategies needed for effective disease management at home. This collaborative approach provided deeper insights into the challenges faced by stroke survivors and their caregivers, ultimately informing better support systems for both groups. Participants who were unable to verbally communicate or provide consent and those who lacked primary caregivers were excluded from the study.

### Data collection

In-depth, semi-structured interviews were conducted to gain insight into the shared experiences of stroke survivors and their caregivers. Dyadic interviews were conducted using open-ended questions to foster collaborative engagement and deeper insights into co-constructed experiences of stroke self-management. This approach was preferred as it enables researchers to observe participant interactions, prompt mutual reflections, and explore how they negotiate shared meanings, uncovering nuanced perspectives that might not surface when interviewed separately [21–23]. During interviews, interaction patterns were noted, and these dynamics were carefully considered during analysis, ensuring that both perspectives were accurately represented and that the findings captured the relational context of stroke self-management.

Interviews took place in locations convenient for participants, with five opting for hospital premises, where a private room was provided, and ten choosing their homes. All interviews were conducted face-to-face between November 28, 2024, and January 31, 2025. One interview was conducted in English, while the remaining interviews were conducted in Twi, the most widely spoken Ghanaian dialect in Ghana, and within the study setting. A translated interview guide and a two-way (forward-back) translation technique were used to ensure accuracy and clarity. Sessions lasted 50–70 minutes and were audio-recorded with participants’ consent. Before the interviews, participants completed a demographic questionnaire covering age, gender, education, and duration of stroke self-management at home. Discussions centered on their experiences with provider support, including satisfaction, unmet needs, and perceived impact on their daily lives. All interviews were conducted by RA, who is fluent in both English and Twi and familiar with Ghanaian cultural norms. To ensure that nuances of meaning, particularly for sensitive topics such as sexuality and emotional distress, were preserved, recordings were transcribed and translated verbatim by a professional transcription service. The forward-back translation approach, along with RA’s linguistic and cultural expertise, facilitated smooth communication and allowed participants to express themselves comfortably in their preferred language. The research team also engaged in cross-checking and reflexive discussions to interpret culturally specific expressions, ensuring that participants’ intended meanings were captured accurately and meaningfully in the analysis.

### Data analysis

Guided by Thorne’s interpretive description methodology, this study employed an inductive approach in which data collection and analysis took place in a continuous and overlapping manner, allowing for ongoing refinement as new data emerged. The analysis unfolded in three overarching stages: (1) organizing and familiarizing with the data, (2) discerning significant patterns, and (3) developing interpretations rooted in participants’ narratives. To begin, RA and EK read the transcripts multiple times to ensure data immersion and to identify recurring ideas and nuances in participants’ experiences. Reflective journaling and annotations helped deepen analytical engagement during this early phase. Emerging insights were documented and initially explored through manual memo writing and highlighting, followed by systematic coding in MAXQDA version 24. Early coding, conducted by RA, centered on noting variations and commonalities across the data. EK, an experienced qualitative researcher, conducted a second coding to ensure consistency. Differences were discussed through iterative dialogue until consensus was reached, and when agreement could not be fully achieved, GK was consulted to provide an independent perspective and help resolve any remaining discrepancies. Codes were then organized into thematic clusters representing participants’ perspectives, and in the second phase, a deeper comparative analysis examined these codes in relation to the study’s core questions. This analytical phase was supported by reflexive notes and the use of constant comparison to uncover connections, distinctions, and evolving meanings across the data set. In the final stage, themes were refined and synthesized to form a coherent narrative that moved beyond surface-level description. Patterns were interpreted in relation to broader contextual factors and theoretical insights, allowing for a nuanced understanding of the experiences under investigation. The interpretive process culminated in findings that not only addressed the research questions but also contributed meaningful implications for healthcare practice and stroke SMS.

### Rigor and reflexivity

The research team comprised male and female stroke researchers, nurses, and nurse specialists with expertise in stroke care and qualitative research. RA, a male nurse with clinical experience in Ghana, conducted the interviews and contributed to data analysis. His professional background and familiarity with the Ghanaian healthcare system facilitated strong rapport with participants, allowing them to share their experiences openly, and supported a culturally informed interpretation of the data. Measures such as reflexive journaling were employed to address potential biases arising from positionality.

To ensure methodological rigor and credibility, this study applied Thorne’s interpretive description approach [18], a qualitative framework well-suited for exploring complex health experiences. Multiple strategies were implemented to uphold trustworthiness across the research process. First, the researcher’s clinical expertise in stroke care provided essential contextual grounding, shaping a coherent interpretive lens informed by both empirical evidence and theoretical insight [18]. Epistemological integrity was reinforced by ensuring alignment among the research question, study design, and analytical process. Representative credibility was strengthened through purposive sampling of individuals with firsthand experience in stroke care and SMS. Data were gathered using semi-structured interviews, which facilitated deep, meaningful dialogue. Establishing rapport and maintaining prolonged engagement supported the authenticity and richness of the narratives [18]. Analytic logic was demonstrated through systematic documentation of research decisions, iterative coding, and pattern recognition following Thorne’s methodological guidelines. Finally, interpretive authority was developed through reflexivity, supported by journaling and constant comparison with existing literature to contextualize and challenge evolving interpretations. These steps contributed to grounded, trustworthy conclusions that reflect the realities of stroke care and SMS in the Ghanaian setting.

## Results

### Characteristics of participants

A total of 30 participants were included in the study, comprising 15 stroke survivors and 15 caregivers. Among the stroke survivors, eight (53.3%) were female, while seven (46.7%) were male. The median age of stroke survivors was 61 years, with a range from 33 to 87 years. In contrast, caregivers were predominantly female (12, 80.0%), with only 3 (20.0%) being male. Caregivers had a median age of 53 years, ranging from 19 to 67 years. All stroke survivors were experiencing their first stroke, with 13 (86.7%) having an ischemic stroke. Participants had been engaged in self-management for a median duration of 8 months, with a range of 2–13 months.

Table 1 provides details of the participants’ characteristics.

**Table 1:** Participants’ Characteristics (N = 30).

Variables	Stroke survivors (n = 15)	Caregivers (n = 15)
**Sex**		
Female	8 (53.3)	12 (80.0)
Male	7 (46.7)	3 (20.0)
**Age median (min-max)**	**61(33-87)**	**53(19-67)**
**Highest level of education**		
No formal education	5 (33.3)	6(40.0) 2(13.3)
Basic education	8 (53.3)	
Secondary education	0 (00.0)	1 (6.7)
Tertiary	2 (13.3)	6 (40.0)
**Stroke Type**		
Ischemic	13 (86.7)	
Hemorrhagic	2 (13.3)	
**Relationship to stroke survivor**		
Spouse		8(53.3)
Child		6 (40.0)
Parent		1 (6.7)
**Self-management duration in months median (min-max)**	8 (2–13)	

Two themes emerged from the analysis: a) unmet educational and support needs for home-based self-management, and b) perceived impact and satisfaction with the support system. Tables 2 and 3 summarize the themes, associated subthemes, and illustrative participant quotations. Table 4 presents the gaps mapped to the respective CCM domains. The identification of gaps followed a systematic and criteria-driven process. Gaps reflect recurring patterns of unmet needs, service deficiencies, and care challenges consistently reported across participant accounts, which are reflected in barriers to continuity, quality, or effectiveness of post-discharge stroke care, and have clear implications for self-management capacity. A deductive mapping process aligned these empirically derived gaps with predefined CCM constructs based on their conceptual definitions and core functions of each CCM domain, ensuring appropriate classification and strengthening the linkage between findings and the CCM framework.

**Table 2:** Summary of unmet educational and support needs for home-based self-management.

Self-management support gaps	Representative quotes
1. Inadequate and inconsistent predischarge education	*“When I was coming home, nobody gave me health education”* (Patient 5) *“They didn’t educate us on what she should eat or do...The only thing they told us was that we should make sure we give us the medication”* (Caregiver 2)
2. Inadequate caregiver training	*“We didn’t receive the needed training to support him in the house. I was expecting them to give us training on how to support him in terms of his movements, exercise, and nutrition in the house”* (Caregiver 14)
3. Lack of ongoing support	*“To be honest, we haven’t received such support. Nobody has ever called us that we are their client, and for that matter, they are checking up on us. We depend on our own experience”* (Caregiver 1) *“Ever since we came home, we have not received any education. I guess they do not care”* (Caregiver 4)
4. Overlooked psychosocial and emotional support	*They didn’t give us any counseling or education on her emotions. I use my own experience. I overlook it when she exhibits those emotional attitudes* (Caregiver 2). *“Some victims of stroke have not given birth before, so when they are caught up in such situations, then it means that they will remain childless. When one is caught up in such a web, one of the couples may cheat. It is worrying”* (Patient 4).
5. Information provision without support	*They didn’t set any goals. They only asked whether we were going for physiotherapy. If they were setting any goals for him, we were not aware because they didn’t tell us about that* (Caregiver 14). *“I need to see some collaboration...somebody should say when you are coming bring this report from the physiotherapy, your progress report and all that. It doesn’t happen. The doctor only asks, ‘Do you go for your physio?’ I was expecting them to say, let’s prepare something for you to give to the doctor for him to see your progress level...but it doesn’t happen”* (Patient 12) *“There isn’t much communication”* (Caregiver 15).

**Table 3:** Summary of the impact of the existing support system on stroke survivors and caregivers and their satisfaction levels.

*Support impact and satisfaction*	Representative quotes
1. Impact of existing support	***a. Experienced some improvement or benefits*** *“The dietary advice has really helped me*” (Patient 1) *“We didn’t see much improvement, so we went for traditional medicine to augment it”* (Caregiver 1).
	***b. Reliance on unqualified individuals for guidance and support*** *“The providers didn’t educate us on mood swings or counsel us on how to deal with mood changes. However, we received advice from people who have experienced stroke”* (Caregiver 15).
2. Patients and caregivers’ satisfaction with the existing support system	**a. Satisfied** *“We are content with the support. We’ve seen improvement. He couldn’t stand or go anywhere, but now, thanks to the care, teachings, and by God’s grace, he is gradually regaining his abilities”* (Caregiver 3).
	**b. Somehow satisfied** *“I realized I was much grateful to the little assistance I received from there. I will be ungrateful if I say they did absolutely nothing to help me. What is quite saddening is the fact that we do not get any assistance now that we are home. I cannot really pinpoint the help we have received now that we are home”* (Caregiver 4)
	**c. Dissatisfied** *“We are not pleased one bit. It is disheartening sometimes”* (Patient 4).

**Table 4:** Identified gaps in post-discharge stroke care mapped to chronic care model domains.

Identified Gaps	Chronic Care Model Domains
Insufficient and inconsistent predischarge education	Self-management support Delivery System Design
Gaps in caregiver training affecting ability to support patient self-management	Self-management support Delivery System Design
Limited continuity of care due to absence of regular follow-ups or home visits.	Self-management support Delivery System Design
Insufficient attention to patients’ and caregivers’ sexual health and emotional needs	Self-Management Support Community Resources and Policies
Limited personalized support addressing unique circumstances and preferences	Self-management support
Absence of educational resources to reinforce self-management information.	Self-management support Delivery System Design
Limited involvement of patients and caregivers in decision-making and goal-setting.	Self-management support
Gaps in collaboration that disrupted continuity of care	Delivery System Design Self-management support Clinical Information System
Communication barriers created by unclear, jargon-filled explanations.	Delivery System Design Self-management support

### Theme 1: Unmet Educational and Support Needs for Home-Based Self-Management

#### Subtheme 1a. Inadequate and inconsistent predischarge education.

Many patients and caregivers described a significant lack of predischarge education essential for effective home-based stroke care. While a few reported receiving some instruction, the majority found the education to be either absent or insufficient. When education was provided, it was often fragmented, inconsistent, and heavily dependent on individual providers’ discretion and expertise. For instance, some patients received general information about their condition, while others were only given selective advice on specific topics such as medication or nutrition. This fragmented approach frequently left families feeling ill-equipped to manage the complex care needs of stroke survivors after discharge. One caregiver captured this shared experience, stating:

*“I would say that we didn’t receive all the necessary education and training to support him at home”* (*Caregiver 12*).

The consequences of this educational gap were, at times, severe. One caregiver recounted a distressing incident that occurred shortly after discharge:

*“Well, we didn’t get much education, so I wouldn’t say they provided education based on our needs. When we came home, he suddenly started bleeding profusely. It was very serious—he almost died...and I didn’t know what to do to stop the bleeding. It was pouring like a tap, and I felt helpless. If we had received proper education on what to expect with stroke and how to handle emergencies, I might have been able to stop the bleeding. Fortunately, we got him to the hospital in time” (Caregiver 14).*

Such experiences highlight the life-threatening implications of inadequate discharge preparation. Beyond emergency response, participants consistently emphasized the need for comprehensive, tailored education on critical components of stroke care, such as nutrition, exercise, mobility, patient handling, and medication management. Many caregivers reported feeling unprepared for both the physical and emotional demands of caregiving, expressing uncertainty about dietary choices, potential drug interactions, and safe medication practices. One caregiver emphasized the need for clearer guidance, particularly around medications:

*“They should have also educated us on her medication—like the reactions of the medicines. Maybe this one should not be mixed with that” (Caregiver 2).*

These findings reflect not only an educational gap but also a systemic issue of inconsistent discharge practices and the absence of standardized, patient-centered education protocols. The burden of learning and adaptation was often placed on families at a time of heightened emotional stress and uncertainty.

#### Subtheme 1b. Inadequate caregiver training.

Beyond the lack of discharge education, many caregivers reported receiving little to no practical training to prepare them for the hands-on demands of caring for stroke survivors at home. While some acknowledged receiving brief skills training, typically focused on tasks like feeding, most noted that essential areas such as mobility assistance, proper positioning, bathing, grooming, and other daily care tasks were either insufficiently addressed or completely overlooked. One caregiver shared:

*“When we were discharged, he couldn’t walk properly, but they didn’t give us any training as to how I could help him” (Caregiver 15).*

Such experiences highlight the significant disconnect between the training caregivers needed and what was actually provided. The lack of comprehensive, skills-based training not only affected caregivers’ confidence and competence but also posed potential risks to patient safety and recovery. These findings underscore the urgent need for structured and holistic caregiver training programs that address the full range of post-stroke care needs to better support both caregivers and stroke survivors in the home setting.

#### Subtheme 1c. Lack of ongoing support.

Participants consistently described a troubling absence of structured, ongoing support following hospital discharge, which left stroke survivors and their caregivers feeling unprepared, isolated, and overwhelmed in managing the recovery process at home. While discharge marked the end of inpatient care, it rarely marked the beginning of meaningful follow-up. Most patients reported receiving no follow-up education, no home visits, and no routine check-ins from healthcare providers to assess their progress, address emerging needs, or reinforce critical self-management practices. One patient captured this systemic oversight plainly:

*“They don’t follow up to see how you are improving—whether I am taking my medicine, whether my blood pressure is high, whether I am eating well, or how I am managing daily activities. None of these things are checked” (Patient 7).*

In the absence of formal follow-up systems, those with financial means sought private healthcare to bridge the gap, revealing stark disparities in access. As one caregiver noted:

*“You have to find a personal doctor to take care of you. I think it’s better to hire a private doctor to provide care at home” (Caregiver 2).*

This dependence on out-of-pocket care highlights the inequities faced by those unable to afford such services. Additionally, the emotional toll of being left to navigate complex care without guidance was deeply felt. These feelings of neglect not only heightened emotional distress but also eroded patients’ confidence in managing their condition effectively. Many caregivers and patients shared a sense of being forgotten once hospital doors closed behind them. One caregiver expressed this sense of abandonment:

*“After leaving the hospital, your fate is in your own hands. Whether you survive or die, nobody cares” (Caregiver 4).*

Participants emphasized the need for consistent and compassionate follow-up, suggesting that even minimal efforts, such as periodic phone calls, can provide reassurance, reinforce accountability, and enhance treatment adherence. They viewed ongoing communication as essential for sustaining motivation, supporting continuity of care, and identifying challenges early. To further ensure adherence, one patient proposed an additional strategy:

*“Occasional, unexpected visits could confirm whether patients are following prescribed treatments” (Patient 5).*

#### Subtheme 1d. Overlooked psychosocial and emotional support.

Participants pointed out a critical gap in stroke care: the absence of guidance and support for the psychosocial and emotional challenges that arise after discharge. Patients and caregivers alike noted that issues related to emotional well-being and sexual function were rarely, if ever, addressed by healthcare providers despite their significant impact on quality of life. Several patients spoke candidly about the changes in sexual function following a stroke, describing feelings of confusion, isolation, and unpreparedness:

*“My libido was completely down in the first few months, but nobody told me this. And you know our system—I mean, certain sensitive cases are difficult for people to discuss” (Patient 12).*

The silence surrounding sexual health left both patients and their partners feeling uncertain and unsupported, with most highlighting their concerns about their unaddressed sexual health challenges and feared potential consequences such as childlessness and infidelity. Despite this, patients and their spousal caregivers were hesitant to voice such concerns for fear of judgment from providers, expecting providers to initiate the discussion:

*“I fear that if I raise that concern, the providers will judge me that my only concern is sex when my husband is sick. So, I am waiting for them to bring up the topic so I can talk about it” (Caregiver 14).*

These accounts underscore the need for healthcare providers to foster safe, open, and nonjudgmental spaces where sensitive topics like intimacy can be discussed without fear or stigma.

Besides sexual health, participants also highlighted the emotional and behavioral shifts that often follow a stroke. Caregivers described feeling overwhelmed by patients’ mood swings, irritability, or emotional outbursts, often without any preparation or coping strategies. One caregiver admitted:

*“Some of the advice should have gone to me as a caregiver to be patient with her. It is difficult dealing with the mood and attitude changes. The way the patient will act, I might be angry to say I won’t take care of her again” (Caregiver 6).*

These emotional burdens left many caregivers feeling unprepared and unsupported, contributing to caregiver strain and potentially affecting the quality of care they could provide. Together, these narratives illuminate a critical gap in stroke recovery support. Addressing the emotional and psychosocial dimensions of post-stroke life, including sexual health, mood changes, and caregiver well-being, is essential for holistic, person-centered care.

#### Subtheme 1e. Information provision without support.

Beyond the lack of structured education and follow-up care, patients and caregivers often received guidance that failed to consider their financial limitations or access to essential resources. While some education was provided, it lacked personalization, failing to address patients’ unique circumstances or offer affordable alternatives. Consequently, many struggled to implement the guidance they received. One patient highlighted the challenge of adhering to dietary recommendations:

*“My eating habit has become distorted because we are told to stay off sugar and salt, everything is monetary. As it stands, I don’t eat meat, and I take advice as given, so I mostly eat smoked fish or dry fish, but I don’t have the financial means. The challenges are too much” (Patient 10).*

Similarly, a caregiver emphasized the need for better nutritional support:

*“When a stroke patient is discharged, the hospital should allocate a dietitian to oversee him. Such a specialist should educate the patient well so that in case he goes home and he doesn’t have money to do certain things, the health education can help improve his health” (Caregiver 1).*

This reflects a missed opportunity to align care with the individual’s financial and social context, an essential aspect of the CCM’s emphasis on personalized care.

Again, participants reported a lack of formal educational materials to support self-management. While verbal instructions were commonly provided, written resources and visual aids, crucial for reinforcing learning and supporting independent care, were largely absent. This posed a significant challenge for both patients and caregivers, who often struggled to retain crucial information. One caregiver shared:

*“They didn’t give us anything except a bill. When we were going home, they didn’t provide any educational material. The dietician was the only one who gave us a copy of her meal routine and advised us to refer to it if we forgot the details. That was the only thing we received”(Caregiver 1).*

The lack of written educational materials contravenes the CCM’s call for accessible, comprehensive resources to support ongoing patient learning and self-management.

Another major concern was the lack of patient and caregiver involvement in decision-making. One caregiver stated,

*“They didn’t set any goals with us” (Caregiver 2),* while another added, *“They haven’t sat down with us to discuss anything” (Caregiver 7).*

This suggests that goal-setting, a core component of effective self-management support, was overlooked. Instead of engaging patients in collaborative discussions, providers primarily delivered instructions without considering patients’ perspectives. As one caregiver described,

*“If you tell them something, they will say you are not supposed to tell them what to do. But if they tell us what we are supposed to do in the form of education, we abide by it” (Caregiver 6).*

This dynamic reflects a one-sided information delivery approach rather than a patient-centered, interactive strategy that empowers patients and caregivers to take an active role in self-management.

This lack of collaboration extended beyond the patient-provider relationship to the broader healthcare team. Participants reported a noticeable disconnect between various providers, especially between physicians and physiotherapists. Rather than coordinating care based on shared information, providers appeared to work in silos, undermining continuity and integrated decision-making. A patient reflected,

*“I expected some collaboration between my doctor and the hospital physiotherapist… But that doesn’t happen. When I see the doctor, they just ask, ‘Do you still go to physiotherapy?’ I say yes, and that’s the end of the conversation” (Patient 12).*

This lack of coordination is a key deviation from the CCM, which stresses the importance of integrated care that ensures all providers are informed and work together towards a common goal.

Compounding these issues was the frequent use of complex medical terminology that alienated patients and delayed critical understanding of their conditions. Participants shared how technical language obscured their diagnoses, leaving them confused and disempowered. One patient recounted,

*“They didn’t tell me I had a stroke… The doctor used medical terms like ‘cerebro something’ and ‘CVA’ in brackets. Until the physiotherapist explained it, we didn’t realize it meant a full-blown stroke. So, the language is a key factor” (Patient 12).*

This failure to use clear, patient-friendly communication contradicts the CCM’s principle of equipping patients with the knowledge they need to be active participants in their care.

These critical gaps in SMS hinder stroke survivors’ empowerment, self-efficacy, and competence in managing their condition at home. Rather than providing comprehensive, patient-centered support, the current approach remains largely informational, failing to align with the interactive, holistic model envisioned in the CCM.

### Theme 2: Perceived Impact and Satisfaction with Support System

#### Subtheme 2a. Impact of existing support.

Despite reports of significant gaps in the existing support system, some participants highlighted some positive impacts of the education received on their health. One patient emphasized the benefits of dietary guidance:

*“The dietary advice has really helped me and has advanced my health status” (Patient 1).*

Caregivers also observed improvements in their loved ones’ conditions. One caregiver shared:

*“The medication and education have helped to control her pressure. Her sugar level is also normal” (Caregiver 8).*

However, the effectiveness of dietary education varied among caregivers. Some did not fully benefit from the nutritional guidance provided and held misconceptions about unhealthy weight gain. As a result, certain caregivers overfed stroke survivors, inadvertently putting their health at risk. One patient shared how his caregiver’s well-intended efforts led to unintended weight gain, which posed additional health concerns:

*“Some people commend me for ‘growing nicely’ because they associate it with good living and quality healthcare from my wife. However, they don’t realize that the more we eat, the more weight we gain, which can negatively impact my health. During checkups, my doctor often comments on my weight, which is largely due to the meals my wife prepares to help me gain a few pounds” (Patient 5).*

Besides this, the lack of comprehensive and continuous support often led stroke survivors and caregivers to seek guidance from unqualified sources, such as friends or other individuals with stroke experience. A recurring concern among participants was the lack of follow-up and ongoing support from healthcare providers, which led some to lose trust in hospital-based care.

Some turned to alternative treatments because they did not see much improvement due to the absence of comprehensive support. A caregiver shared:

*“We didn’t see much improvement, so we went for traditional medicine to augment it” (Caregiver 1).*

Contrarily, some patients adopted a proactive approach to understanding their health in response to the lack of formal education and support. Literate patients and caregivers often took the initiative to educate themselves or drew upon their existing knowledge rather than waiting for professional guidance. Others turned to family members, friends, or peers with similar health experiences as alternative sources of information and support. One patient shared:

*“Loved ones who visit me are those that encourage me not to be anxious” (Patient 4).* Another participant shared,

*“Those with similar situations rather gave us education” (Caregiver 6).*

#### Subtheme 2b. Patients’ and caregivers’ satisfaction with the existing support system.

Participants expressed mixed feelings about the adequacy of support provided by the healthcare system, with a prevailing sentiment of dissatisfaction due to gaps in follow-up care and comprehensive education. While some acknowledged improvements and minimal assistance, many felt that the support system was insufficient and failed to meet their expectations. Several caregivers and patients voiced strong dissatisfaction, citing a lack of continuous support and follow-up. One caregiver emphasized the need for a more comprehensive approach:

*“The support was not enough because I think that if a person is suffering from stroke, they should receive comprehensive education and support, but they didn’t give us that” (Caregiver 2).*

Similarly, a patient acknowledged systemic shortcomings but accepted the status quo:

*“We are not satisfied with that, but we will take it like that because that is the usual practice in Ghana and it’s not as if I am being treated differently” (Patient 2).*

Another participant expressed frustration:

*“We are not pleased one bit. It is disheartening sometimes” (Patient 4).*

While a small number of participants acknowledged some level of improvement, the majority expressed dissatisfaction with the existing support system. The most pressing concerns included the absence of follow-up care, inadequate education, financial barriers to seeking alternative care, and the emotional burden of managing conditions without sufficient professional support. These findings highlight the need for improved patient follow-up, better home-care guidance, and more comprehensive education to enhance patient and caregiver experiences.

In Table 4, identified gaps are mapped to the CCM domains based on the nature of the reported deficiencies and their conceptual alignment with each domain’s core functions. Gaps related to patient education, caregiver preparedness, individualized support, patient engagement, and reinforcement of self-management behaviors indicate limited capacity to empower stroke survivors and caregivers to manage post-discharge care effectively, reflecting weaknesses in the Self-Management Support domain. Deficiencies in follow-up care, fragmented service delivery, poor coordination, and communication barriers highlight weaknesses in the organization and continuity of care, corresponding to limitations in the Delivery System Design domain. Challenges in interprofessional communication, coordination across care settings, and transfer of patient information reflect breakdowns in information exchange processes, indicating deficiencies within the Clinical Information Systems domain. Emotional, psychosocial, and sexual health concerns point to insufficient psychosocial support and limited integration with external supportive services, such as counseling and peer support, reflecting gaps in both the Self-Management Support and Community Resources and Policies domains. Thus, this mapping demonstrates a systematic alignment between identified care gaps and the functional responsibilities of the CCM domains.

## Discussion

This study explored the current SMS practices in Ghana to assess the support stroke survivors and their caregivers receive from healthcare providers for home-based management after discharge. It is one of the first studies in Ghana to systematically examine SMS for stroke survivors through the lens of the CCM and incorporating the perspectives of both patients and caregivers. Findings of the study revealed that the predischarge preparation received by stroke survivors and their caregivers focused primarily on education and skills training. Despite this, patients and caregivers voiced concerns that these efforts were often limited to didactic, informational content, lacking interactivity and patient-centered engagement. This approach falls short of the CCM, which emphasizes that effective SMS should extend beyond education and skill-building; it should embrace a proactive, culturally sensitive, and collaborative approach that empowers patients to manage their conditions with confidence.

Beyond reflecting an educational gap, the findings highlight broader systemic challenges, including inconsistent discharge practices and the absence of standardized, patient-centered protocols. This inconsistency shifts the burden of learning and adaptation to families during a period marked by emotional distress and uncertainty. Again, patients and caregivers reported limited opportunities for hands-on, practical training tailored to their unique needs, particularly in managing mobility, medication safety, and emergency preparedness.

To align with the CCM and achieve effective SMS, predischarge education must be structured, comprehensive, and culturally tailored*.* It should go beyond one-size-fits-all approaches by actively involving caregivers in the discharge process and equipping them with practical, role-specific training. The absence of such support undermines caregivers’ confidence and competence, increasing the risk of preventable complications, including falls, bed sores and aspiration among stroke survivors [24,25]. These issues also contribute to higher rates of hospital readmissions and poor long-term recovery outcomes [26]. Therefore, health systems in Ghana should prioritize the development and implementation of standardized predischarge education programs that include simulation-based caregiver training, culturally sensitive communication strategies, and structured follow-up support. Integrating these elements into discharge planning aligns with the SMS and delivery system design CCM components and can significantly improve confidence, reduce preventable complications, and enhance the success of home-based stroke recovery. Additionally, healthcare providers should view caregivers not as peripheral supporters, but as co-clients and active participants in the care process. This requires offering them targeted education and meaningful support to strengthen their role in long-term care.

Patients and caregivers also reported that they did not receive structured educational materials or programs to adequately support them. The lack of sufficient resources and the failure to prioritize interactive and ongoing education as core elements of patient care significantly hinder their ability to manage the condition at home. Without accessible, user-friendly educational materials, stroke survivors and caregivers face considerable challenges in disease management. This lack of support results in decreased patient engagement, motivation, and overall confidence in self-management. To address these gaps, healthcare facilities should develop and offer structured, easy-to-use educational materials and incorporate ongoing, interactive teaching into routine care.

Another significant concern was the absence of structured follow-up care and continuous support after discharge. The absence of essential follow-up measures, such as timely check-in calls and home visits, results in missed opportunities to monitor patient progress, identify emerging challenges, and address evolving care needs. In this study, many stroke survivors and caregivers, in an attempt to cope with the lack of formal support, turned to alternative treatments and unqualified individuals. This trend is common in Ghana and has been reported by several studies [11,27]. While this shift may reflect adaptive coping strategies aimed at filling a care gap, it also introduces considerable risks, including misinformation, delayed engagement with evidence-based interventions, harmful drug interactions, and compromised safety [28,29]. Implementing structured follow-up programs, including routine home visits and check-in calls as well as technology-driven innovation programs such as tele- and mobile-health interventions, to monitor patient progress, identify challenges, and provide ongoing education and support in resource-limited settings like Ghana, could enhance post-discharge care, promote sustained SMS, and significantly improve stroke outcomes and caregiver well-being [30].

A notable concern raised by participants was the lack of coordination among healthcare providers in post-discharge stroke care. This fragmentation points to gaps in the delivery system design domain, disrupting continuity of care and compromising treatment decisions, thereby undermining the CCM’s emphasis on coordinated team-based care. Without collaboration, providers operate in silos, which prevents the creation of personalized and cohesive SMS strategies. Consequences of this fragmentation include the omission of crucial medical history, repeated diagnostic testing, and the administration of unnecessary medicine. Moreover, patients and caregivers are often excluded from decision-making and collaborative goal-setting processes, which are key components of patient-centered care. This exclusion not only weakens engagement but also creates a disconnect between clinical goals and patient priorities, further complicating recovery [31].

The use of medical jargon also emerged as a significant barrier for patients and caregivers in this study, impairing their ability to understand the diagnosis and delaying critical actions needed for effective self-management and recovery. Prior studies have shown that heavy reliance on medical jargon causes confusion and can make providers seem unapproachable or uncaring, eroding trust and discouraging communication [32,33]. This can limit patient engagement and worsen outcomes. To address this, healthcare providers should adopt patient-centered communication strategies, using plain language, visual aids, written handouts, and simple analogies to explain complex information. These approaches not only improve communication and trust but also contribute to greater patient satisfaction and better health outcomes.

Besides these, concerns were raised regarding the neglect of sexual health needs among stroke survivors. Spousal caregivers, in particular, reported feeling unable to discuss these issues due to fear of judgment, as providers failed to create an open and supportive environment for sensitive conversations. Consequently, many patients were left without crucial information, support, or treatment. This finding aligns with studies suggesting that sexual health concerns are often overlooked in healthcare settings due to provider discomfort, misaligned provider-patient priorities, or insufficient provider training in addressing such issues effectively [34]. Comprehensive SMS should address psychological issues, including sexual health, alongside physical health. Studies have reported outcomes such as depression and diminished quality of life with overlooking such concerns [35], highlighting the need to integrate sexual health into stroke rehabilitation.

Again, stroke survivors and caregivers expressed dissatisfaction with the counseling and support provided to address emotional and behavioral challenges. This shortcoming contradicts the CCM, which emphasizes comprehensive SMS encompassing emotional and behavioral aspects of chronic disease management. The CCM stresses the importance of collaborative care, proactive interventions, and a multidisciplinary approach to support patients and caregivers in managing the psychological and emotional impact of conditions like stroke. The absence of such well-structured support can lead to increased stress, anxiety, depression, caregiver burnout, hospital readmissions, and poor self-management outcomes [36].

Finally, this study examined patients’ and caregivers’ perceptions of post-discharge support from healthcare providers and their satisfaction with the support received. While some participants acknowledged minor improvements, dissatisfaction was the prevailing sentiment due to gaps in follow-up care and inadequate education. Key concerns included insufficient follow-up, limited patient education, financial barriers to accessing care, inadequate connection to essential resources, and a lack of collaborative goal-setting. Additionally, participants reported inadequate support for emotional and sexual health needs, compounding the emotional burden of managing chronic conditions with minimal professional support. These findings underscore the urgent need for a more comprehensive post-discharge support system that aligns with the CCM to enhance patient and caregiver experiences.

Notably, financial constraints emerged as a significant barrier to care, consistent with previous research attributing this issue to the high out-of-pocket expenses and the limited scope of Ghana’s NHIS [37]. These financial challenges impede access to essential services, including medications, follow-up care, and rehabilitation, thereby undermining recovery and the effective long-term management of chronic conditions. Addressing these systemic barriers necessitates a critical evaluation and expansion of the NHIS benefits package to provide more comprehensive coverage for chronic disease care. In parallel, implementing cost-effective strategies, such as mobile health technologies, may help bridge service delivery gaps by facilitating remote support, health education, and timely follow-up care [38,39]. Collectively, these efforts have the potential to promote health equity, enhance home-based chronic disease management, and ultimately improve health outcomes across Ghana.

### Strengths and limitations

This study was conducted in a single Ghanaian hospital, which may limit the transferability of findings to other institutions due to variations in protocols, resource distribution, and local practices. While the hospital shares similarities with other public facilities, broader insights could be gained through multi-site or national studies. Additionally, participants recalled self-management support experiences a median of eight months post-discharge, introducing potential recall bias. Their perceptions may have shifted over time, affecting the accuracy of reported experiences. Future prospective longitudinal studies could better capture the evolving support needs of stroke survivors.

#### Implications for Practice and Policy.

This study highlights critical deficiencies in the support available to stroke survivors and their caregivers for self-management at home. Ghana’s healthcare system lacks an integrated SMS framework within its chronic care framework, leading to inadequate prioritization, commitment, and resource allocation for post-discharge care. Addressing these gaps requires urgent policy reforms to enhance predischarge preparation, follow-up care, and ongoing support. Implementing structured post-discharge programs, such as telemedicine check-ins or home visits by community health nurses, could significantly improve patient outcomes. Strengthening collaboration between hospitals and community healthcare services is also essential, ensuring coordinated care through interdisciplinary partnerships involving stroke care teams, primary care providers, and home-based caregivers. Additionally, healthcare professionals should receive periodic specialized training in SMS strategies to equip them with the skills needed to support patients and families in long-term stroke management. The study further emphasizes the need for tailored interventions to address the psychological, social, and financial challenges of stroke survivors and caregivers. Furthermore, advocating for policy changes to secure sustainable funding for follow-up care is essential. Stable financial support would empower healthcare providers to deliver comprehensive, patient-centered care, ensuring stroke survivors receive the ongoing support needed for effective self-management and improved health outcomes.

## Conclusion

Stroke remains a major public health concern in Ghana, with survivors often facing significant challenges in managing their condition following hospital discharge. This study identified several gaps in the support offered to stroke patients and their caregivers, highlighting a broader systemic challenge with the integration of chronic care into healthcare systems. A key area of concern was the shortcomings in the continuity of care. Although the CCM presents a useful lens for improving chronic disease management, its principles are not yet fully embedded within Ghana’s post-stroke care landscape. The findings underscore the urgent need to develop patient- and caregiver-centered interventions, policies, and resources that strengthen home-based support systems. Addressing these gaps can empower stroke survivors and caregivers to manage recovery more effectively, ultimately enhancing long-term health outcomes and quality of life.

## Supporting information

S1 Appendix Semi-structured interview guide. Interview guide used to explore the experiences of discharged stroke survivors and their caregivers regarding the self-management support they received from healthcare providers following hospital discharge.
(DOCX)
