# Supplementary material for: Stroke patients and caregivers’ experiences and satisfaction with post-discharge self-management support in Ghana: A qualitative study
Source: PLOS Glob Public Health. 2026 Jun 26;6(6):e0006702. doi: 10.1371/journal.pgph.0006702 (PMC13309047; doi:10.1371/journal.pgph.0006702)
Supplement: S1 Appendix — Interview guide used to explore the experiences of discharged stroke survivors and their caregivers regarding the self-management support they received from healthcare providers following hospital discharge. (DOCX) [file pgph.0006702.s001.docx]

**APPENDIX 1**

**Interview Guide**

Let’s start with a few questions about you.

**Introductory questions**

1. How do you want to be referred to? (Your first name or pseudonym)
2. Can you tell us your age?
3. How do you identify yourself?
4. Male

ii. Female

1. What is your highest education?
2. How long have you been diagnosed with stroke?
3. How long have you been discharged from the hospital?

**SECTION A – The Current Healthcare Self-Management Support in Ghana**

Let’s talk about the current healthcare SELF-MANAGEMENT SUPPORT in Ghana.

Let's start by chatting about the kind of support you received while you were in the hospital and after going home.

1. **Before you were discharged from the hospital**, what kind of preparation or support did you and your caregiver get? Did the doctors or nurses offer any education, training, or counseling to help you manage the stroke at home?
2. Did the support you received meet your expectations?

**If Yes**

1. In what ways did it meet your expectations?
2. How has that support helped with managing the stroke at home? Do you think it has had an impact on your health and quality of life?
3. Is there anything you would have liked to see improved in the preparation you got before going home?
4. **If is No**
5. How do you feel the lack of preparation affected managing the stroke at home?
6. What challenges did you face because of this?
7. What do you think could be done differently to improve the pre-discharge support for people managing stroke at home?
8. **Since you’ve been home**, what kind of support have you and your caregiver received from doctors or nurses? This could include things like education, counseling, or home visits.
9. Are you satisfied with the post-discharge support you’ve been getting for self-management at home?

**If Yes**

- 1. How does the support you're getting meet your expectations?
  2. How has this support helped with managing the stroke, and what effect has it had on your quality of life?
  3. Do you have any suggestions on how healthcare professionals could improve home-based self-management?

**If No**

1. How has this affected your ability to manage the stroke at home and your overall health?
2. What specific challenges or gaps have you noticed in the support you’ve been getting?
3. What improvements would you suggest to make the self-management support more effective?
4. What educational and discharge materials did you receive from the hospital or healthcare providers when you were discharged?

**SECTION B – The Concept of Healthcare Self-Management Support**

Now, I’d love to hear your thoughts about what kind of support would be most helpful.

1. To help stroke survivors have better health outcomes and quality of life, in your opinion:
2. **Before going home,** what kind of preparation or resources do you think would have been helpful for you and your caregiver to better manage the stroke at home?
3. **After discharge**, what kind of ongoing support do you think stroke survivors and their families need from healthcare professionals?

**SECTION C – Providing a Standardized Healthcare Self-Management Support in Ghana: Barriers and Facilitators**

Let’s discuss some of the challenges and helpful aspects of the healthcare support you’ve received.

1. Thinking about the support you’ve received both before and after going home:
   1. Before discharge, did healthcare providers assess your needs to determine the kind of support or resources you might need to manage the stroke at home?

**If yes,** how did that assessment affect the preparation or support you received?

**If not,** how did not having that assessment affect your preparation for managing the stroke at home?

- 1. Before going home, did you feel that you received all the education, training, and resources you needed for self-management?

**If yes,**

What constitutes this preparation?

How did this preparation impact your health and quality of life?

**If not,**

How did you manage without it, and how has it affected your health and quality of life?

- 1. After discharge, did you continue to get the necessary education and training to manage the stroke at home?

**If yes,** what kind of training and education have you received, and how has it helped?

**If not**, how have you been managing without that continued support?

- 1. Do healthcare providers work with you to set goals and monitor your progress in managing the stroke at home?

**If yes,** who are the team members involved, and how does their support affect your health?

**If not,** how do you deal with challenges on your own, and how has this affected your self-management and quality of life?

1. Are you assigned a specific healthcare professional to coordinate your care after discharge? Do they follow up or visit you at home?

**If yes**, how often do they contact or visit you, and how has this impacted your recovery and well-being?

**If not,** how is your follow-up care managed and who do you reach out to if you need help?

1. Have you received support to help improve your self-care ability and mobility, like devices or rehabilitation?

**If yes,** what kind of support did you get, and how has it affected your health and independence?

**If not,** how have you managed, and how has this impacted your health?

1. Are you getting support to help you reintegrate into your community, like returning to work or participating in social activities?

**If yes,** what specific help have you received, and how has this affected your quality of life?

**If not,** how are you managing, and how has this affected your ability to engage in social activities or work?

1. Have you received support or resources to help deal with the emotional changes that come with having a stroke?

**If yes,** how often do you receive this support, and how has it affected your health?

**If not,** how do you cope with these emotional changes?

1. Have you received support for dealing with behavioral changes caused by the stroke?

**If yes,** how often do you receive this help, and how has it impacted your quality of life?

**If not,** how do you manage these changes?

1. Have you received support for dealing with sexual function changes caused by the stroke?

**If yes**, how often do you receive this help, and how has it impacted your quality of life?

**If not,** how do you manage these changes, and how does it affect your quality of life?

1. Do you feel that the education and training provided by healthcare professionals are clear and easy to understand?

**If yes**, how has this affected your ability to manage the stroke?

**If not,** what challenges do you face in understanding or following their guidance?

1. Are you given enough time to absorb the information you’re being taught, and do healthcare professionals repeat key points or encourage you to take notes?

**If yes,** how has this helped you manage the stroke?

**If not,** how has the lack of this affected your management and health?

1. Do healthcare professionals encourage and support you to build confidence and motivation to manage your condition at home?

**If yes,** how has this affected your ability to manage the stroke?

**If not,** how has the absence of this support impacted your recovery?

**Summary**

Before we wrap up, let’s go over the key points we’ve discussed. Is there anything you’d like to add or clarify?

Thank you so much for your time and insights!
